# Effect of culture relatedness on affective neural responses to words in L1 and L2: ERP studies

**DOI:** 10.3389/fnhum.2026.1762627

**Published:** 2026-08-04

**Authors:** Yanxi Lu, Bo Yao, Kate Cain, Francesca M. M. Citron

**Affiliations:** Department of Psychology, Lancaster University, Lancaster, United Kingdom

**Keywords:** bilinguals, Chinese, culture, emotional valence, English, ERPs

## Abstract

This study investigates affective responses to words in bilinguals’ native (L1) and second (L2) languages. Specifically, we examine how the extent to which concepts are related to one’s own native or second culture influences these emotional responses. While previous research has shown that emotional responses are often attenuated in the L2 compared to the L1, the role of culture in this phenomenon remains underexplored. Using event-related potentials (ERPs), we recorded neural responses from 34 Mandarin-English bilinguals during valence judgment tasks involving emotionally positive or neutral words. The words were either related to Chinese culture, e.g., *rice*, British culture, e.g., *scones*, or unrelated to either culture, e.g., *sake*, and were either positive or neutral in valence, e.g., *lotus flower* vs. *chopsticks.* Each participant completed experiments in both their L1 (Mandarin) and L2 (British English). Behavioral results revealed faster reaction times for positive words and significant interactions between emotional valence and culture relatedness, in both L1 and L2. ERP analyses focused on two key components: the early posterior negativity (EPN), associated with early emotional attention, and the late positive potential (LPC), reflecting sustained affective engagement. In the L2, a significant main effect of valence was observed on the EPN. In contrast, no main effect of valence was found in the L1; instead, significant main effects of culture relatedness were observed on both the EPN and LPC components. Our results suggest that culture relatedness can influence the affective processing of words and modulate neural responses and provide new evidence that culture relatedness shapes bilinguals’ emotional experiences at both behavioral and neural levels, above and beyond the well-established overall “emotional distance” from L2.

## Introduction

Given the ubiquity of multilingual communication, understanding how bilinguals navigate emotional content is critical. However, research on multilinguals has disproportionately prioritized the investigation of cognitive mechanisms, leaving affective dimensions largely underexplored. The present paper investigates how cultural context modulates affective responses to a second language (L2) This focus moves beyond traditional research on bilinguals’ language processing, which has primarily centered on cognitive aspects, such as semantic and syntactic differences between the first language (L1) and the L2, and their underlying neural representations. Previous research has addressed a broad scope of topics, including syntactic prediction (e.g., Hartsuiker and Pickering, 2008), speech production (e.g., Costa et al., 2005), and the debate between parallel activation of both languages and automatic L1 translation (e.g., Costa et al., 2017; Oppenheim et al., 2018; Thierry and Wu, 2007). In contrast, the affective processing of bilinguals has received comparatively less attention, although a growing body of work now investigates how emotional content is experienced and processed in both languages. Early psychophysiological studies demonstrated that emotionally charged expressions often elicit stronger autonomic responses in the first language than in the second, with these differences influenced by factors such as age of acquisition and learning context (Harris et al., 2006). However, behavioral findings have not always been consistent. For example, Eilola et al. (2007) reported similar interference effects for negative and taboo words in late bilinguals’ L1 and L2 during an emotional Stroop task, indicating that language differences in affective processing may depend on the specific task and the type of emotional material examined.

Recent decades have witnessed a pivotal shift toward examining the affective underpinnings of bilingual language processing. A recurring proposal in this literature is that of “emotional distance” or reduced emotional resonance in the L2, namely the idea that affective content in a later-acquired language may evoke weaker or less immediate emotional responses than in the L1. That is, emotional material in the L2 may exert a smaller impact on behavior and may elicit attenuated or delayed physiological and neural responses relative to the L1 (e.g., Pavlenko, 2012). Researchers have used a range of methodologies to investigate this possibility, including psychophysiological measures, EEG/ERPs, and fMRI. Importantly, physiological evidence has often provided some of the clearest support for this account. For instance, Toivo and Scheepers (2019) showed that high-arousing words elicited stronger pupillary responses in bilinguals’ L1 than in their L2, even when lexical variables were carefully controlled. Taken together, these findings suggest that reduced emotional resonance in the L2 is a robust but not uniform phenomenon, whose manifestation may vary across measures, task demands, and characteristics of the bilingual experience.

Building on these findings of reduced emotional resonance in the L2, subsequent research has begun to explore potential moderating factors. Some behavioral studies have suggested that such weakened affective processing may be partially reduced through improvements in L2 proficiency or increased language dominance. Degner et al. (2012) used an affective priming task to assess whether the emotional valence of L2 words was activated automatically, that is, whether affective congruency between a prime and a target influenced responses even when emotion was not the explicit focus of the task. They found reliable affective priming in the L1, whereas in the L2 it emerged only in bilinguals with greater language immersion and more frequent L2 use. Imbault et al. (2021) collected emotional valence and arousal ratings for English words from non-native speakers of English living in Canada, and found that higher proficiency and longer time residing in Canada were associated with smaller differences in emotional ratings when compared to native speakers. Nevertheless, the mechanisms underlying emotional distance in L2 processing remain only partially understood. Existing research suggests that factors such as proficiency, language dominance, frequency of L2 use, and length of residence in an L2 environment may reduce L1–L2 differences in affective responding. However, these findings have mainly focused on language-experience variables, and it remains unclear whether they are sufficient to explain emotional distance more broadly, or how other factors, such as cultural experience and cultural congruence, may contribute to it.

The relationship between language and culture has been highlighted across disciplines, including psychology and linguistics (Hoijer, 1954). In second language education, it is widely accepted that acquiring an L2 involves not only learning its linguistic elements but also internalizing its cultural context (e.g., see Kramsch, 2014, for a review on applied linguistics and language education). Svanes (1987) reported that cultural distance predicted second-language achievement more strongly than integrative motivation. In that study, cultural distance did not refer to a single factor, but to a broader constellation of variables, including learners’ familiarity with Western culture, the linguistic distance between Norwegian and their native language, and their proficiency in other European languages, including English. By contrast, integrative motivation refers to learners’ interest in, and willingness to identify or engage with, the target-language community and culture. Thus, the findings suggest that broader cultural-linguistic proximity to the target context may, in some cases, be more predictive of L2 outcomes than motivation alone.

Empirical evidence also suggests that cultural context influences communication in the L2. Zhang et al. (2013) tested Mandarin-English bilinguals living in the United States by presenting them with culturally salient images—for example, Chinese icons such as the Great Wall and American icons such as Mount Rushmore—and then asking them to describe these images in English and to narrate stories from a separate set of culture-neutral pictures. Exposure to Chinese, relative to American, cultural icons reduced English speech fluency, as reflected in lower listener-rated fluency and slower speech rate, not only when participants described the cultural images themselves but also when they later told stories about culture-neutral pictures. These findings suggest that cultural context can interfere with L2 performance, even when the speaking task is not itself culturally themed.

In the context of L2 language and cultural adaptation, Wright (2000) found that the affective approach, i.e., encouraging students to relate personal experiences to L2 culture, was more effective than merely providing cultural information through textbooks. Neuroscientific evidence also indicates that culture affects emotional processing. For instance, East Asian culture emphasizes emotional restraint compared to Western cultures, which tend to encourage explicit emotional expression. Murata et al. (2013) used EEG to measure emotional simulus processing in East Asians and Euro-Americans. They found that only East Asians showed reduced late positive potential (LPP) when instructed to suppress emotions, suggesting that East Asians are culturally conditioned to suppress emotions more effectively than Westerners.

Neuroimaging techniques have also been used to examine culture effects on brain activity. From a cross-cultural psychological perspective, people’s experiences shape their brains, and these experiences can vary depending on cultural background. Neuroimaging studies have provided evidence for culture effects on neural activity, particularly those that reflect cognitive functions (e.g., Han and Northoff, 2008). For example, Morrison et al. (2003) found that, while participants showed similar brain activation when listening to culturally familiar and unfamiliar music, they performed better when recognizing culturally familiar music. Ng et al. (2010) found that individuals with bicultural backgrounds exhibited brain activity patterns more consistent with the culture primed during a task. Park and Huang (2010) reviewed evidence that culture affects neural function, noting differences between East Asian collectivistic and Western individualistic cultural contexts.

However, the mechanisms underlying cultural influences on bilingual affective processing remain poorly understood, particularly regarding how cultural congruence between content and language might modulate emotional responses. In the present study, we selected emotionally positive and neutral words that are related to Chinese or British culture as well as words unrelated to either culture. In two experiments, we presented Mandarin-British English bilinguals with these words, written in Mandarin in one experiment and in English in the other experiment. We asked participants to judged their emotional valence (i.e., whether it was positive or neutral) while recording their EEG activity as well as type of responses (positive vs. neutral) and reaction times.

Given the existing literature, it is reasonable to hypothesize that culture influences affective processing in bilingual contexts. For bilinguals, cultural context may determine the degree of emotional engagement in either L1 or L2. For instance, bilinguals may feel more emotionally engaged with content related to their native culture, regardless of the language in which it is presented, therefore showing larger amplitude of emotion-related ERP components for Mandarin culture-related words than either British culture-related words or unrelated words. Alternatively, engagement with L2 cultural content might enhance emotional resonance in the L2 due to the adoption of new cultural norms, therefore showing similar amplitude of emotion-related ERP components in L1 and L2, larger for L1 and L2 culture-related words than non-related words. In addition, in line with literature on emotion word processing, we predict positive words to be read faster and to show larger amplitude or emotion-related ERP components compared to neutral words (e.g., Citron, 2012; Sereno et al., 2015). Finally, we have no specific predictions with regards to reading speed in response to words with difference cultural references.

Despite these intriguing predictions, little empirical work has explored how culture and language together shape affective processing in bilinguals. This study aims to address this gap by systematically investigating how cultural context and language congruence interact to shape affective processing in bilinguals, with implications for understanding the fundamental nature of emotion-language-culture relationships. With this work, we hope to provide deeper insights into the intricate relationship between culture, emotion, and language processing.

## Method

A preregistration of this study is published and available at: https://aspredicted.org/m64hk.pdf.

### Participants

A total of 34 Lancaster University students aged between 19 and 35 (6 men, *M* = 25.2 years) were recruited through campus poster advertisements. All participants were late, unbalanced Mandarin-English bilinguals who had spent most of their time in China before the age of 18. They had achieved a CEFR level of C1 or above in English proficiency. With one exception who self-identified as Mongolian Chinese, all participants self-identified as Han Chinese. Each participant consented to participation and received a compensation of £22.5. All participants were right handed, neurotypical, with no reported history of neurological or psychiatric disorders and had normal or corrected-to-normal vision.

### Materials

#### Preparatory norms

Two preparatory norming studies were conducted, one on Mandarin words and one on English words. Each study used a questionnaire to collect ratings for familiarity, valence, culture relatedness to British culture, culture relatedness to Chinese culture and imageability, for a list of words in the corresponding language. For the English questionnaire, a set of 600 words was sourced from two main categories: 400 words were selected from the English norms of Warriner et al. (2013), and 100 words were translated from Mandarin norms with reference to Xu et al. (2022). The remaining 100 words or word pairs were commonly known British culture items, including food (e.g., Fish and Chips), locations (e.g., Lancaster), and well-known names (e.g., Princess Diana). For the Mandarin questionnaire, which included a set of 450 words, 150 words were selected from Mandarin norms with reference to Xu et al. (2022), 150 words were translated from the English norms of Warriner et al. (2013), and the other 150 words or word pairs were commonly known British culture items, including food (e.g., 炸鱼薯条, Fish and Chips), locations (e.g., 兰卡斯特, Lancaster), and well-known names (e.g., 黛安娜王妃, Princess Diana).

Participants rated each dimension on a 7-point scale. Valence was rated on a bipolar scale ranging from −3 (*very negative*) to +3 (*very positive*), with 0 indicating *neutral* (*neither positive nor negative*). Familiarity was rated from 0 (*I never come across it*) to 6 (*I come across it all the time*). Imageability was rated from 0 (*extremely difficult to imagine*) to 6 (*extremely easy to imagine*). Culture relatedness was rated separately for Chinese culture and British culture, each on a scale from 0 (*not at all related to Chinese/British culture*) to 6 (*highly related to Chinese/British culture*).

Emotional valence and arousal are both well-established affective variables that influence language processing (Russell, 1980; Russell, 2003). However, although arousal is an important affective dimension, it was not included as a stimulus-selection variable in the present study. We prioritized valence because it is generally more stable and has been more consistently linked to behavioral and neural measures in emotion word research, whereas arousal ratings tend to be more variable across participants and are often better captured through physiological indices. Accordingly, for the purposes of the present ERP experiment, stimuli were selected and matched on valence, familiarity, imageability, and culture relatedness.

In each norming study, instructions in the according language were given before participants started rating each attribute. Participants first rated familiarity, followed by emotional valence. Subsequently, they rated imageability and culture relatedness (to Chinese and British cultures). The imageability rating was presented between the two culture relatedness rating tasks, which were counterbalanced across participants (i.e., half rated relatedness to Chinese culture first, and half rated relatedness to British culture first). The order in which participants rated each attribute was carefully considered to minimize the potential influence of order effects. In detail, participants rated their level of familiarity (or subjective frequency of encounter) with the words first. As participants will encounter each word five times in the questionnaire, rating their familiarity at the very beginning can mitigate potential confusion stemming from encountering the words repeatedly. After rating familiarity, participants were instructed to rate the valence of the words, without being explicitly informed that they would rate culture relatedness in the next stage. In other words, cultural considerations were not brought to the attention of the participants to ensure that valence ratings of the words were not significantly influenced by their salient cultural considerations when rating the words. After rating familiarity and valence, half of the participants rated culture relatedness to Chinese culture, followed by imageability, and then culture relatedness to British culture. Accordingly, the other half of participants rated culture relatedness to British culture, followed by imageability, and then culture relatedness to Chinese culture. Imageability was rated in between to avoid transfer effects between the two cultures and also because its ratings are based on meaning and are therefore less likely to be influenced by ratings of other variables. In the end, each word received at least 10 valid ratings for each attribute. These ratings were obtained from participant samples that were entirely independent of the ERP sample reported in the present study. In addition, prior to formal rating, a prescreening step was conducted to verify the cultural appropriateness of candidate items. English items were prescreened by one English monolingual speaker and one bilingual speaker with English as L1 and Mandarin as L2. Mandarin items were prescreened by two bilingual speakers with Mandarin Chinese as L1 and English as L2.

#### Visual stimuli for Study 1

A set of 240 English words or word pairs were selected for use in the EEG experiment based on the preparatory norms. These stimuli were allocated to following conditions: Valence (positive vs. neutral) and Culture (related to British culture, related to Chinese culture, or unrelated to either culture). We decided to focus exclusively on positive and neutral valence, as our initial screening indicated that negative concepts rarely satisfied the strict criteria for cultural specificity required for our design. To ensure comparability, word stimuli in the six conditions were matched for word length (i.e., number of letters for English words, and number of characters for Mandarin words), rated familiarity and rated imageability. Additionally, we matched valence ratings across the three cultural conditions, ensuring that positive words were equally positive and neutral words equally neutral, regardless of their culture relatedness (see Table 1 for descriptive statistics).

**Table 1:** Descriptives for English words by culture and valence condition.

Condition: valence	Condition: culture	Valence		Familiarity		Imageability		Length	
		*M*	*SD*	*M*	*SD*	*M*	*SD*	*M*	*SD*
Neutral	British	0.39	0.23	3.83	0.92	4.32	0.85	8.95	3.78
	Chinese	0.38	0.35	3.68	0.78	4.23	0.72	9.15	2.94
	Unrelated	0.40	0.30	3.85	0.83	4.22	0.73	9.05	2.00
Positive	British	1.00	0.22	3.96	0.65	4.24	0.63	9.08	4.35
	Chinese	1.08	0.35	3.76	0.84	4.50	0.58	9.20	4.21
	Unrelated	1.05	0.30	3.90	0.78	4.36	0.78	9.10	1.72

Valence was rated on a Likert Scale from −3 (extremely negative) to 3 (extremely positive); Familiarity was rated on a Likert Scale from 0 (not at all familiar) to 6 (extremely familiar); Imageability was rated on a Likert Scale from 0 (not at all easy to imagine) to 6 (extremely easy to imagine).

As a result, among the selected words, valence ratings showed significant differences across distinct valence conditions [*F*(1, 234) = 289.77, *p* < 0.001, *ηₚ^2^* = 0.553]. However, valence ratings remained consistent across various culture conditions [*F*(2, 234) = 0.56, *p* = 0.912, *ηₚ^2^* = 0.001], and the interaction between valence and culture was not statistically significant [*F*(2, 234) = 1.64, *p* = 0.197, *ηₚ^2^* = 0.014].

For culture relatedness to Chinese culture, ratings differed significantly across culture conditions [*F*(2, 234) = 490.83, *p* < 0.001, *ηₚ^2^* = 0.808]. No significant differences were observed across valence conditions [*F*(1, 234) = 2.37, *p* = 0.125, *ηₚ^2^* = 0.010], and no interaction between valence and culture was observed [*F*(2, 234) = 0.40, *p* = 0.671, *ηₚ^2^* = 0.003].

Similarly, for culture relatedness to British culture, ratings differed significantly across culture conditions [*F*(2, 234) = 336.64, *p* < 0.001, *ηₚ^2^* = 0.742]. No significant differences were observed across valence conditions [*F*(1, 234) = 1.14, *p* = 0.286, *ηₚ^2^* = 0.005], and no interaction between valence and culture was observed [*F*(2, 234) = 0.88, *p* = 0.415, *ηₚ^2^* = 0.007].

For familiarity ratings, no significant differences were observed across valence conditions [*F*(1, 234) = 0.30, *p* = 0.594, ηₚ^2^ = 0.001] or culture conditions [*F*(2, 234) = 1.81, *p* = 0.166, *ηₚ^2^* = 0.015], and no significant interaction was observed, [*F*(2, 234) = 0.12, *p* = 0.891, *ηₚ^2^* < 0.001].

Similarly, imageability ratings showed no significant differences across valence conditions [*F*(1, 234) = 2.58, *p* = 0.109, *ηₚ^2^* = 0.011] or culture conditions [*F*(2, 234) = 0.09, *p* = 0.912, *ηₚ^2^* = 0.001], and no significant interaction, [*F*(2, 234) = 1.64, *p* = 0.197, *ηₚ^2^* = 0.014].

Finally, word length in letters did not significantly differ across valence conditions [*F*(1, 234) = 0.03, *p* = 0.862, *ηₚ^2^* < 0.001] or culture conditions [*F*(2, 234) = 0.05, *p* = 0.953, *ηₚ^2^* < 0.001], and no significant interaction was found either, [*F*(2, 234) = 0.00, *p* = 0.997, *ηₚ^2^* < 0.001].

#### Visual stimuli for Study 2

In a similar manner, 240 Mandarin Chinese words or word pairs were chosen for utilization in the EEG experiment, guided by the preliminary norms. These stimuli were displayed using a font size of 18 and the SongTi font. The allocation of these stimuli followed specific conditions: Valence (positive vs. neutral) and Culture (associated with British culture, linked to Chinese culture, or not linked to either culture). To ensure consistency, the word stimuli were balanced in terms of rated familiarity, visual complexity, and rated imageability. Visual complexity was determined by the cumulative count of strokes in simplified Chinese characters (简体字). Additionally, the valence ratings of words were balanced within the same valence condition, and matched across different levels of culture-relatedness (see Table 2 for descriptive statistics).

**Table 2:** Descriptives for Mandarin words by culture and valence condition.

Condition: valence	Condition: culture	Valence		Familiarity		Imageability		Visual complexity	
		*M*	*SD*	*M*	*SD*	*M*	*SD*	*M*	*SD*
Neutral	British	0.15	0.25	3.12	0.63	4.30	0.89	21.90	8.34
	Chinese	0.19	0.28	3.03	0.65	4.44	0.88	21.43	6.30
	Unrelated	0.20	0.23	3.01	0.75	4.20	0.89	22.85	7.10
Positive	British	0.97	0.32	3.18	0.66	4.39	0.76	23.80	8.67
	Chinese	0.96	0.34	3.04	0.84	4.37	0.65	22.80	9.33
	Unrelated	0.91	0.37	3.03	0.60	4.49	0.74	23.45	7.04

As a result, among selected words, valence ratings significantly differed across different valence conditions, *F*(1, 234) = 333.65, *p* < 0.001*, ηₚ^2^* = 0.588. Meanwhile, valence ratings were consistent across different culture conditions, *F*(2, 234) = 0.26, *p* = 0.770, *ηₚ^2^* = 0.002. There was no significant interaction between valence and culture on valence ratings, *F*(2, 234) = 0.08, *p* = 0.923, *ηₚ^2^* = 0.001.

For culture relatedness to Chinese culture, no significant differences were observed across valence conditions [*F*(1, 234) = 0.52, *p* = 0.472, *ηₚ^2^* = 0.002]. Ratings differed significantly across culture conditions [*F*(2, 234) = 451.46, *p* < 0.001, *ηₚ^2^* = 0.794], and no significant interaction between valence and culture was observed [*F*(2, 234) = 0.27, *p* = 0.763, *ηₚ^2^* = 0.002].

Similarly, for culture relatedness to British culture, no significant differences were observed across valence conditions [*F*(1, 234) = 0.11, *p* = 0.736, *ηₚ^2^* < 0.001]. Ratings differed significantly across culture conditions [*F*(2, 234) = 537.96, *p* < 0.001, *ηₚ^2^* = 0.821], and no significant interaction between valence and culture was found [*F*(2, 234) = 1.29, *p* = 0.278, *ηₚ^2^* = 0.011].

As for familiarity ratings, no significant differences was found across valence conditions, *F*(1, 234) = 0.00, *p* = 0.989, *η*ₚ^2^ < 0.001, or culture conditions, *F*(2, 234) = 0.61, *p* = 0.544, *η*ₚ^2^ = 0.005, and no significant interaction, *F*(2, 234) = 0.05, *p* = 0.948, *η*ₚ^2^ < 0.001.

Similar to familiarity, imageability ratings showed no significant differences across valence conditions, *F*(1, 234) = 1.40, *p* = 0.238, *η*ₚ^2^ = 0.006, or culture conditions, *F*(2, 234) = 0.30, *p* = 0.744, *η*ₚ^2^ = 0.003, and no significant interaction, *F*(2, 234) = 0.93, *p* = 0.397, *η*ₚ^2^ = 0.008.

Lastly, visual complexity (i.e., cumulative stroke count) did not differ significantly among valence conditions, *F*(1, 234) = 1.62, *p* = 0.205, *η*ₚ^2^ = 0.007, or culture conditions, *F*(2, 234) = 0.37, *p* = 0.692, *η*ₚ^2^ = 0.003, and no significant interaction was found, *F*(2, 234) = 0.14, *p* = 0.871, *η*ₚ^2^ = 0.001.

### Procedure

#### Language history questionnaire (10 min)

Participants first completed the Language History Questionnaire (LHQ3, Li et al., 2020), remotely and online, providing information on their language background, including their age of acquisition and competence in all of the languages with what they had been in contact.

#### Lab task and EEG recording (120 min)

Participants came to the lab to take part in two distinct experiments, one involving 240 Chinese words or word pairs and the other involving 240 English words or word pairs. The two stimulus sets were constructed separately within each language and were not designed as translation equivalents, though some word or word pairs may have overlapped semantically across the two experiments. The order of the two experiments was counterbalanced among participants, with a minimum 5-min break, which could be extended based on their preferences, before moving on to the next experiment, to reduce potential priming effects. Within each experiment, the words of six conditions were presented in pseudo-randomized order using four presentation lists that were distributed evenly across participants, with the additional constraint that no more than two trials from the same valence condition appeared consecutively.

The task for both experiments was identical: participants needed to subjectively respond to the valence category (positive vs. neutral) of any presented word or word pair, using the left key (index finger) or the right key (middle finger) on a keyboard. We counterbalanced the response mapping across participants: half of the group used the left key for positive responses, while the other half used the right. However, to minimize confusion, we kept this mapping consistent for each individual participant across both their Mandarin and English sessions. Also, we counterbalanced the sequence of conducting Mandarin and English testings for each participant.

During each experiment, participants were comfortably seated in front of a computer monitor, positioned at a consistent distance of 80 cm from participants’ eyes. The experiments were programmed using PsychoPy (v2022.2.4, Peirce et al., 2019). At the outset of each experiment was the practice, where participants were instructed to categorize two words into “neutral” or “positive”; these words were not part of the main stimulus set. Following the practice, the experimental trials started, each sequentially showing a prompt image as a reminder for blinking, a fixation cross, and a word or word pair (i.e., stimulus). In each trial, prior to the presentation of the stimulus, participants were prompted to blink when a picture of eyes appeared, which lasted for 1,500 ms. Then, participants were prompted to fixate their gaze on the fixation cross (white, line width = 1) that appeared at the center of the screen, for a variable duration ranging from 500 ms to 900 ms. We utilized this procedure to prevent participants from blinking during the word classification. Following the fixation cross, a word was presented at the center of the screen, until the participant made a keyboard response or for a maximum duration of 2,500 ms, whichever was shorter. Stimuli in both Study 1 (English) and Study 2 (Mandarin Chinese) were displayed in Open Sans with height = 0.03 and the window units set to height. Given the experiment window size of 1,440 × 900 pixels, this corresponds to an approximate on-screen character height of 27 pixels or roughly 20 pt. in conventional word-processing terms. The word was followed by a blank screen that persisted until 3,500 ms after the onset of the word stimuli. Type of responses and reaction times (RTs) in milliseconds were recorded.

For each experiment, EEG signals were continuously recorded using 64 electrodes placed on the scalp, while additional sets of bipolar electrodes were applied around participants’ left eye to detect blinking and horizontal eye movements. We recorded neural activity using a 64-channel BrainProducts EEG system (Brain Products GmbH, Gilching, Germany). We utilized a BrainAmp amplifier to acquire data at a sampling rate of 500 Hz and used standard 64-channel ActiCAPs with head circumferences ranging from 54 to 60 cm. To ensure optimal conductivity, we applied SuperVisc high-viscosity electrolyte gel during setup and maintained electrode impedances at 5 kΩ or less. Signal quality was monitored throughout the sessions using BrainVision Recorder software (Brain Products GmbH, Gilching, Germany).

#### Valence rating questionnaire (25 min)

After finishing the EEG task, participants were asked to rate the valence of the word stimuli shown during the experiments in an online questionnaire. Despite efforts assuring the valence ratings only had significant differences between the two valence conditions (positive vs. neutral), the tasks essentially examine participants’ subjective interpretation of word valence. Since the valence ratings were gathered from a different participant sample (though with the same characteristics as the participants in this research), different individuals might have different personal perceptions of the words. Therefore, comparing the valence ratings from the same participant sample of the EEG experiments to the valence ratings used for stimulsu selection can confirm the validity of the norms collected and thus aid in the interpretation of any observations in the EEG data.

### Data analysis

Based on the literature, we identified two ERP components of interest in our dataset, both related to the processing of emotive content of words (as well as images): the early posterior negativity, EPN (200–300 ms, temporo-occipital scalp distribution), and late positive component, LPC (500–800 ms, centro-parietal scalp distribution; Citron, 2012; Hinojosa et al., 2020). In our specific studies, based on the observation of the ERPs, the electrodes selected for the EPN were: P7, P8, O1, O2, PO4, PO7, PO8 (the neighboring electrode PO3 was not included because it showed comparatively poorer data quality and greater noise in the present dataset), and the time window was between 100 and 280 ms for both Study 1 (English words, L2) and Study 2 (Mandarin words, L1); the electrodes selected for the LPC were: Cz, C1, C2, CPz, and the time window was between 500 and 700 ms for Study 1 (English words, L2), and between 420 and 620 ms for Study 2 (Mandarin words, L1).

The selection of electrodes and decision of time windows were guided both by prior literature and by inspection of the ERP waveforms during preprocessing, independently of the condition-level statistical analyses. Especially, the onset of EPN is earlier than the range commonly reported for the EPN. The grand-average waveforms in the present dataset showed a posterior negative-going effect beginning at approximately 100 ms and continuing into the more typical EPN interval. We therefore selected the 100–280 ms window to capture the full morphology of this posterior negativity, while acknowledging that its early onset should be interpreted cautiously.

For each participant in each study, and for each stimulus presented, we extracted the mean amplitude of the EPN across all selected electrodes, and the mean amplitude of the LPC across all selected electrodes. For each study, and for each ERP component of interest, linear mixed-effects models (LMMs) were employed to explore the effects of culture-relatedness and emotional valence, as well as their interaction, on neural responses. The dependent variable was the mean ERP amplitude, with fixed effects including culture-relatedness, emotional valence and their interaction. Participants were modeled as a random intercept to account for subject-level variability. The same model was conducted to analyze RTs as dependent variable. A generalized Imixed-effects model with the same predictors was instead used to analyze the type of response (positive vs. negative). Given the relatively small final ERP samples, we adopted a parsimonious random-effects structure to ensure stable model estimation.

These studies are exploratory. However, expected meaningful interactive patterns could be, for example, larger amplitude differences between positive and neutral Chinese-culture-related words compared to the difference found in British-culture-related or unrelated words. Alternatively, positive Chinese-culture-related words may exhibit a larger amplitude of emotion ERP components than positive British-culture-related or unrelated words. Any main effect of culture-relatedness will also be reported. The main effect of culture-relatedness is not the primary focus, as differences between the three conditions may be due to familiarity or processing difficulty. No claims of stronger affective engagement can be made without comparing emotive vs. neutral stimuli. In summary, the primary interest is to investigate potential interactions between the three cultural conditions and the two emotional valence conditions. Nevertheless, any main effects will also be reported.

## Results

### Behavioral results

#### Reaction times (RTs)

Tables 3, 4 below show the descriptives of RTs (ms) for L2 words (Study 1, English) and L1 words (Study 2, Mandarin) respectively. Descriptive statistics for both studies suggest that RTs tend to be shorter for positive words than for neutral words within corresponding culture conditions. Meanwhile, RTs were considerably longer for the L2 words compared to the L1 words.

**Table 3:** Descriptive statistics for RTs (ms) in Study 1 - English words (L2).

Valence	Culture	Reaction times (ms)		
		*n*	*M*	*SD*
Positive	British	1,292	1319.96	456.31
	Unrelated	1,305	1319.62	456.46
	Chinese	1,287	1402.81	506.54
Neutral	British	1,295	1355.45	452.95
	Unrelated	1,309	1393.18	455.70
	Chinese	1,300	1425.06	485.02
Total		7,788	1369.35	470.80

*n* represents the number of valid responses within each group; *M* = mean; *SD* = standard deviation.

**Table 4:** Descriptive statistics for RTs (ms) in Study 2 - Mandarin words (L1).

Valence	Culture	Reaction times (ms)		
		*n*	*M*	*SD*
Positive	British	1,294	1028.17	391.11
	Unrelated	1,298	977.47	380.92
	Chinese	1,304	1001.90	375.89
Neutral	British	1,298	1047.35	398.07
	Unrelated	1,295	1055.80	403.65
	Chinese	1,301	1044.46	404.48
Total		7,790	1025.83	393.35

*n* represents the number of valid responses within each group; *M*, mean; *SD*, standard deviation.

For each study, RTs were log-transformed to improve normality. For each participant, log-transformed RTs exceeding ± 2.5 standard deviations (*SD*s) were excluded from the analysis. Afterwards, if a participant’s RT data contained fewer than 30 valid observations in any of the six conditions (3 culture x 2 valence), all data from that participant were excluded from the analysis.

**Figure 1:**
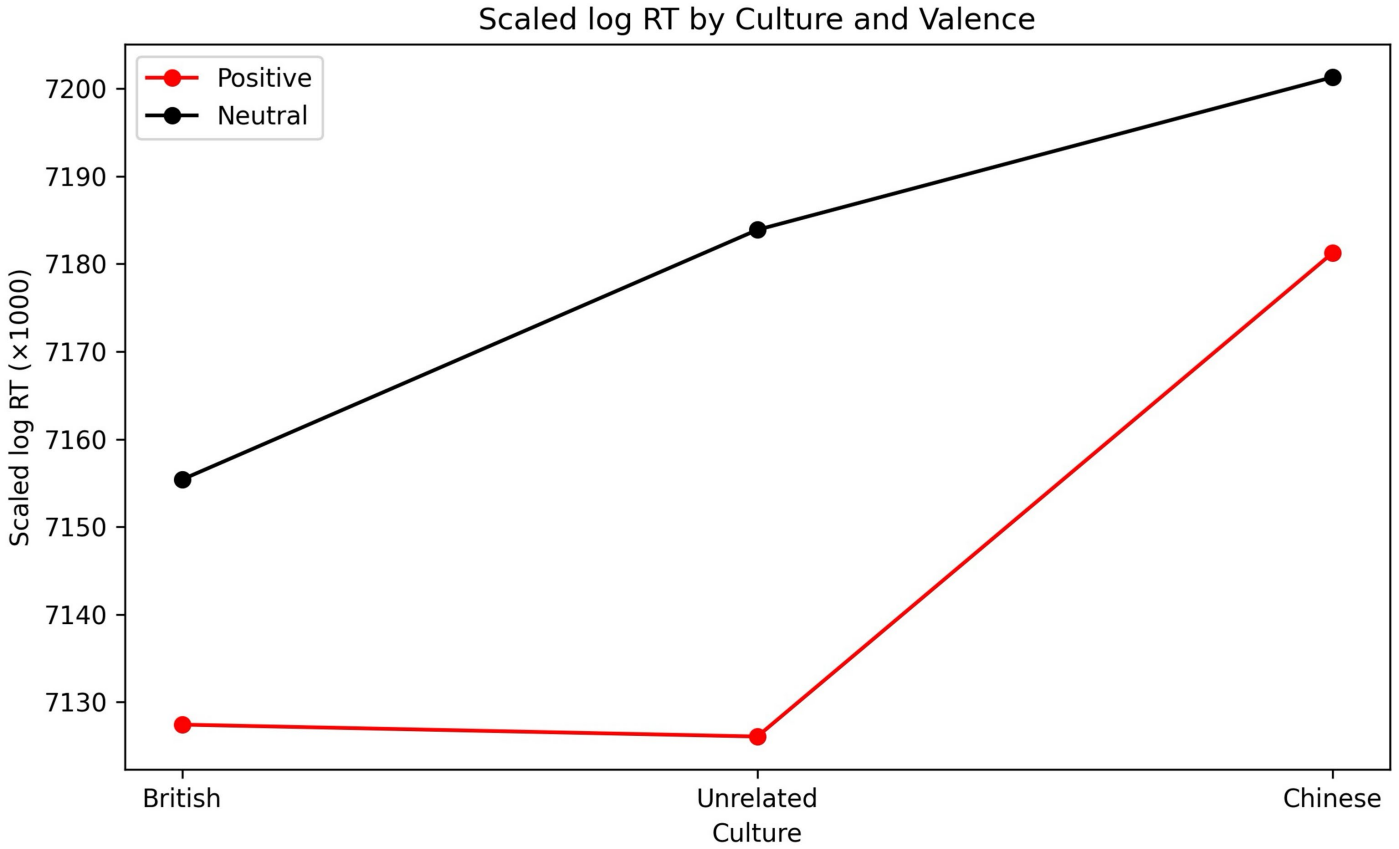
Mean log RTs by culture relatedness and valence in Study 1 – English (L2). Values represent log-transformed RTs multiplied by 1,000 to improve readability.

Table 5 and Figure 1 illustrate the results of LMM analyses for the L2 words. A significant effect of valence on log-transformed RTs was observed. Specifically, participants responded more quickly to positive words than to neutral ones (*β* = −0.06, *p* < 0.001). Significant interactions occurred between valence and the two culture-related conditions (British and Chinese). The results indicated that, for positively valenced words, participants took longer to judge the valence when words were related to British (*β* = 0.03, *p* = 0.041) or Chinese culture (*β* = 0.04, *p* = 0.016) compared to unrelated words. Unexpectedly, a main effect of British culture was also present (*β* = −0.03, *p* = 0.009), showing faster RTs to words related to British than unrelated words. The model included participant-level random intercepts. The estimated variance of the random intercept was 0.04. The model yielded a marginal *R^2^* of 0.007 and a conditional *R^2^* of 0.300.

**Table 5:** LMM analysis: culture relatedness and valence effects on Log RTs in Study 1 – English (L2).

Predictor	*β*	*SE*	*CI*	*p*
Intercept	7.18	0.034	[7.117, 7.251]	<0.001***
British	−0.03	0.012	[−0.053, −0.076]	0.009**
Chinese	0.02	0.012	[−0.05, 0.397]	0.137
Positive	−0.06	0.012	[−0.081, −0.362]	<0.001***
British × Positive	0.33	0.016	[0.013, 0.065]	0.041*
Chinese × Positive	0.04	0.016	[0.074, 0.713]	0.016*

*SE*, standard error of the mean; CI, 95% confidence interval. When analyzing the effect of cultural salience, the reference was set to be the unrelated condition. Significance thresholds: * *p* < 0.05, **p < 0.01, ***p < 0.001.

**Figure 2:**
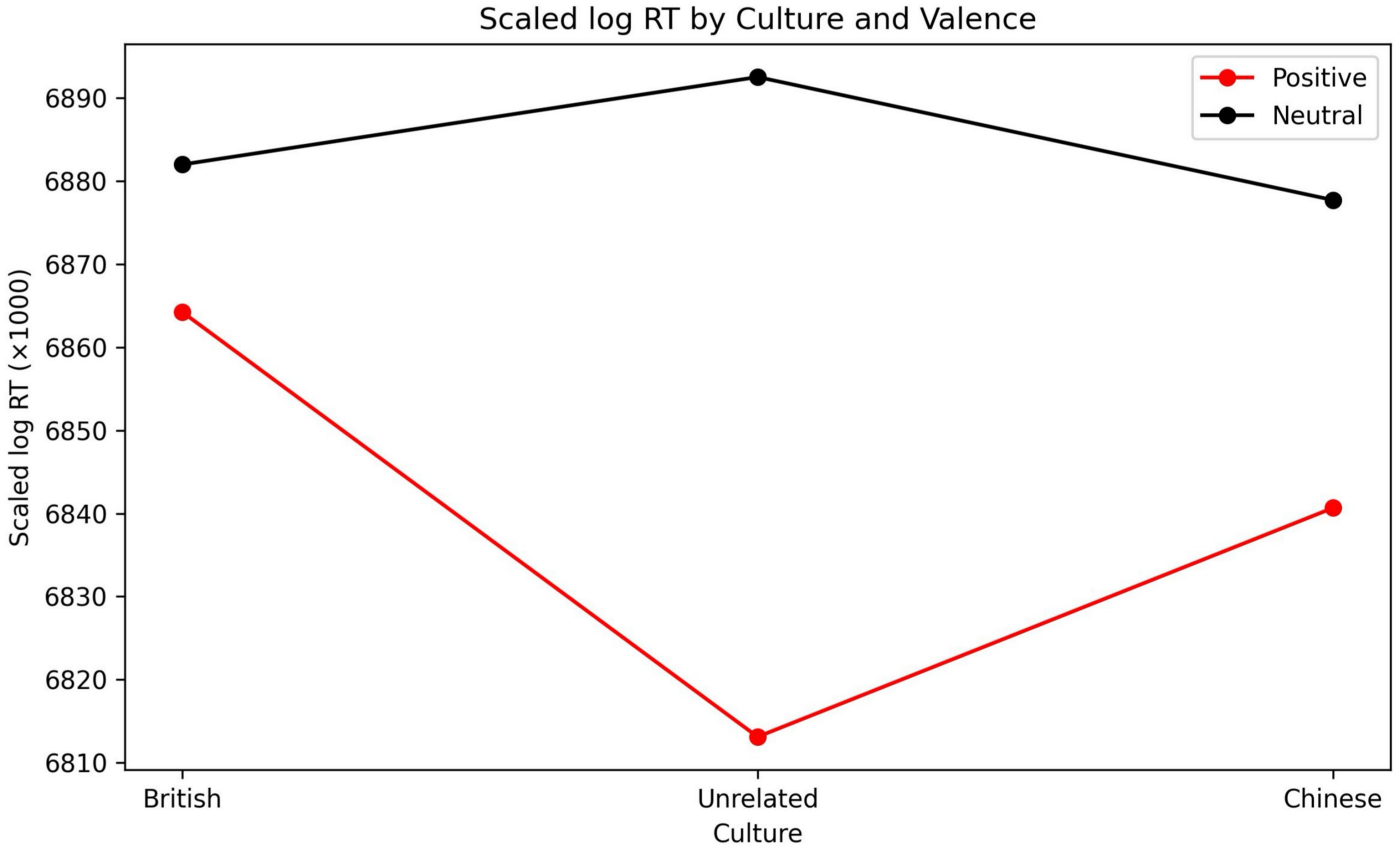
Mean Log RTs by Culture relatedness and Valence in Study 2 - Mandarin (L1). Note. Values represent log-transformed RTs multiplied by 1,000 to improve readability.

Table 6 and Figure 2 present the results of the LMM analysis for the L1 words. The results were generally similar to those of the L2 words, except that, unlike in the L2, no main effect of culture was observed. Participants responded more quickly to positive words than to neutral ones (*β* = −0.08, *p* < 0.001). Significant interactions between valence and the two culturally related conditions (British and Chinese) were observed. In more detail, participants took longer to judge the valence for the positive words when they were related to British (*β* = 0.06, *p* < 0.001) or Chinese culture (*β* = 0.04, *p* = 0.007) than for unrelated words. The model included participant-level random intercepts. The estimated variance of the random intercept was 0.07. The model yielded a marginal *R^2^* of 0.005 and a conditional *R^2^* of 0.489.

**Table 6:** LMM analysis: culture relatedness and valence effects on Log RTs in Study 2 – Mandarin (L1).

Predictor	*β*	*SE*	CI	*p*
Intercept	6.89	0.05	[6.80, 6.98]	<0.001***
British	−0.010	0.01	[−0.03, 0.01]	0.355
Chinese	−0.01	0.01	[−0.03, 0.08]	0.228
Positive	−0.08	0.01	[−0.10, −0.06]	<0.001***
British × Positive	0.06	0.02	[0.031, 0.09]	<0.001***
Chinese × Positive	0.04	0.02	[0.011, 0.07]	0.007*

*SE*, standard error of the mean; CI, 95% confidence interval. When analyzing the effect of cultural salience, the reference was set to be the unrelated condition. Significance thresholds: * *p* < 0.05, **p < 0.01, ***p < 0.001.

#### Type of response

Due to the subjective nature of the task involving valence decisions, there were no correct or incorrect responses. Tables 7, 8 demonstrate the distribution of types of responses for each condition.

**Table 7:** Type of responses in Study 1 - English words (L2): descriptive statistics.

Valence	Culture	Count of responses			
		*n*	Positive	Neutral	Congruency
Positive	British	1,292	731	561	57%
	Unrelated	1,305	817	488	63%
	Chinese	1,287	804	483	62%
Total		3,884	2,352	1,532	61%
Neutral	British	1,295	604	691	53%
	Unrelated	1,309	547	762	58%
	Chinese	1,300	571	729	56%
Total		3,904	1722	2,182	56%
Overall total		7,788	4,074	3,714	58%

*n*, no. of valid responses. The ‘Congruent’ column refers to the percentage of responses that match the valence condition.

**Table 8:** Type of responses in Study 2 - Mandarin words (L1): descriptive statistics.

Valence	Culture	Count of responses			
		*n*	Positive	Neutral	Congruency
Positive	British	1,294	867	427	67%
	Unrelated	1,298	939	359	72%
	Chinese	1,304	933	371	72%
Total		3,896	2,739	1,157	70%
Neutral	British	1,298	625	673	52%
	Unrelated	1,295	613	682	53%
	Chinese	1,301	648	653	50%
Total		3,894	1886	2008	52%
Overall total		7,790	4,625	3,165	61%

*n*, no. of valid responses. The ‘Congruency’ column refers to the percentage of responses that match the valence condition.

To examine whether response congruency exceeded chance level, we conducted one-tailed binomial tests against a chance level of 50% for each condition in both L1 and L2. In the L2, congruency was significantly above chance in all conditions. For both positive and neutral words, congruency exceeded chance across all three culture conditions, and the same held when responses were collapsed across culture conditions. In the L2, congruency was significantly above chance for all positive-word conditions and for positive words overall. For neutral words, however, congruency was significantly above chance only for unrelated items, whereas British-culture-related and Chinese-culture-related neutral items did not differ significantly from chance. When collapsed across culture conditions, overall congruency for neutral words was nevertheless significantly above chance.

Logistic regression analyses were performed for both datasets. As shown in Tables 9, 10, for both L1 and L2 a significant effect of positive valence on the type of response was found. Specifically, positive valence significantly increased the likelihood of positive responses (*β* = 1.07, *p* < 0.001 in L1; *β* = 0.85, *p* < 0.001 in the L2), thereby validating the valence conditions. However, the interaction between British culture relatedness and positive valence decreased the probability of positive responses (*β* = −0.29, *p* = 0.014 in the L2; *β* = −0.45, *p* < 0.001 in the L2), i.e., positive words related to British culture were judged as positive less often than words related to Chinese culture or unrelated. Of greater concern was the significant main effect of British culture relatedness in the L2 (*β* = −0.45, *p* < 0.001), i.e., words related to British culture were judged as positive more often than words related to Chinese culture or unrelated, potentially explaining the unexpected significantly faster RTs for words related to British culture.

**Table 9:** Logistic regression analysis: culture relatedness and valence effects on type of response in Study 1 - English (L2).

Predictor	*β*	*SE*	CI	*p*
Intercept	−0.33	0.06	[−0.441, −0.222]	<0.001***
British	0.20	0.08	[0.042, 0.352]	0.013*
Chinese	0.09	0.08	[−0.068, 0.242]	0.270
Positive	0.85	0.08	[0.690, 1.004]	<0.001***
British × Positive	−0.45	0.11	[−0.668, −0.227]	<0.001***
Chinese × Positive	−0.09	0.11	[−0.315, 0.129]	0.412

Neutral responses were recoded as 0, and positive responses were recoded as 1. *SE*, standard error of the mean; CI, 95% confidence interval. When analyzing the effect of culture relatedness, the reference was set to be the unrelated condition. Significance thresholds: *p < 0.05, **p < 0.01, ***p < 0.001.

**Table 10:** Logistic regression analysis: culture relatedness and valence effects on type of response in Study 2 - Mandarin (L1).

Predictor	*β*	*SE*	CI	*p*
Intercept	−0.11	0.06	[−0.216, 0.002]	0.055
British	0.03	0.08	[−0.121, 0.187]	0.678
Chinese	0.10	0.08	[−0.055, 0.253]	0.208
Positive	1.07	0.08	[0.905, 1.232]	< 0.001***
British × Positive	−0.29	0.12	[−0.514, −0.058]	0.014*
Chinese × Positive	−0.14	0.12	[−0.368, 0.092]	0.239

Neutral responses were recoded as 0, and positive responses were recoded as 1. *SE*, standard error of the mean; CI, 95% confidence interval. When analyzing the effect of culture relatedness, the reference was set to be the unrelated condition. Significance thresholds: *p < 0.05, **p < 0.01, ***p < 0.001.

### ERP results based on planned analyses

**Figure 3:**
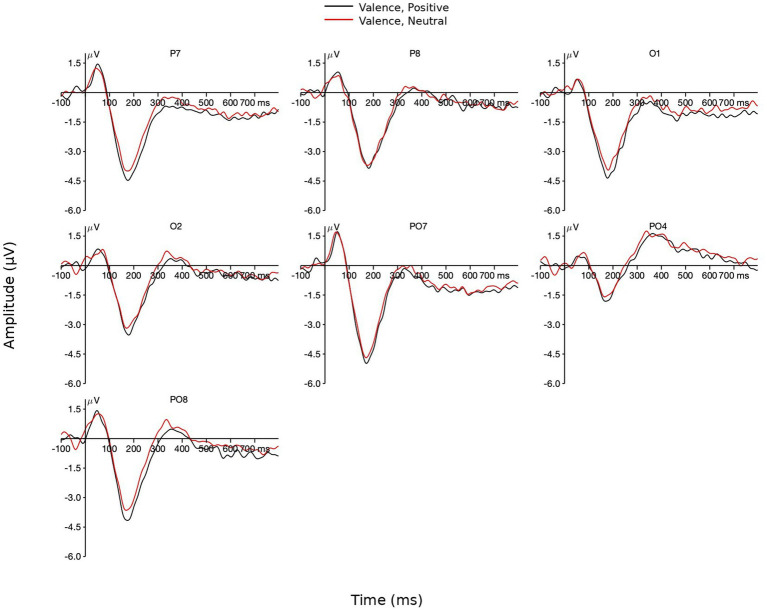
Mean ERPs for valence conditions (positive vs. neutral) on electrodes selected for the EPN component (see Data Analysis in the Method section) of English words. For the statistical analyses, a time window between 100 and 280 ms was used.

**Figure 4:**
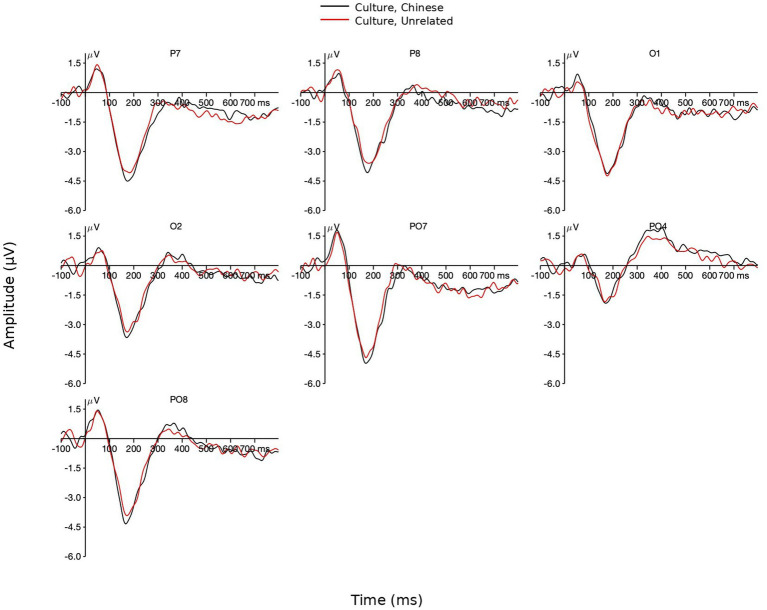
Mean ERPs for two culture conditions (Chinese vs. unrelated) on electrodes selected for the EPN component (see data analysis in the method section) of English words. For the statistical analyses, a time window between 100 and 280 ms was used.

Plotting of the ERP data and visual inspection showed clear EPN and LPC components, each of them displaying typical scalp distribution and latencies that are in line with the literature (see Figures 3, 4).

#### EEG preprocessing

All EEG data preprocessing was conducted in MATLAB using EEGLAB (Delorme and Makeig, 2004) and ERPLAB (Lopez-Calderon and Luck, 2014). For each participant, raw EEG files were imported, and channel types were assigned. Data were high-pass filtered at 1 Hz and low-pass filtered at 30 Hz. Line noise was removed using the CleanLine plugin (Mullen, 2012) with harmonics up to 250 Hz, and bad channels were automatically detected and removed using the clean_flatlines and clean_channels functions. Removed channels were then interpolated using spherical spline interpolation based on the original channel montage. After restoring EOG channels, the data were re-referenced to the average of all scalp electrodes. All preprocessing steps and statistics, including the number of interpolated channels and remaining epochs, were logged automatically for each participant. Epochs were extracted time-locked to stimulus events of interest from −1 to 2 s. Independent component analysis (ICA) was performed on epoched data with data rank reduction as appropriate. ICA components were automatically classified and flagged using ICLabel (Pion-Tonachini et al., 2019); artifactual components were identified using standard thresholds. The number of artifact-related components for each participant was recorded for quality control. Epochs of −100 to 800 ms relative to stimulus onset were extracted for ERP analysis. All 34 participants completed both Study 1 (English, L2) and Study 2 (Mandarin, L1), and the order of the two studies was counterbalanced across participants. There was a 15-min break between the two tasks, during which impedance was checked and, where necessary, electrodes were adjusted while keeping the cap in place. Although both datasets were collected within the same EEG session for each participant, data quality varied across the two studies for the same individual. Therefore, EEG preprocessing and participant inclusion were conducted separately for Study 1 and Study 2.

Two participants were excluded from both studies because of poor signal recording quality. For the remaining datasets, channels with inter-channel correlation below 0.80 were interpolated, and any dataset requiring interpolation of more than 15% of channels (i.e., 10 or more channels) was excluded from ERP analysis. Applying this criterion resulted in 19 usable datasets for Study 1 and 24 usable datasets for Study 2. A further trial-retention criterion was then applied after artefact rejection: participants were required to retain at least two-thirds of trials in each condition (i.e., at least 27 trials per condition). After this step, the final ERP sample comprised 17 participants for Study 1 and 18 participants for Study 2.

For Study 1 with English (L2) words, the statistical analyses of ERPs included 3,746 rials across 17 participants (*range* = 188–237 trials per participant). For Study 2 with Mandarin (L1) words, the statistical analyses of ERPs included 4,007 trials across 18 participants (*range* = 207–238 trials per participant). All the participants completed both studies 1 and 2, and the participants’ sequence of completing the tasks of studies 1 and 2 was counterbalanced: half of the participants took Study 1 (English) first, while the rest of them took Study 2 (Chinese) first. There was a 15-min break between Study 1 and 2, during which necessary re-setup and/or maintenance work (i.e., the electrodes remained on the initial set-up place on participants’ scalp during the break, while we conducted impedance check and accordingly re-setup some of electrodes) was done.

Under this condition, each participant’s results of studies 1 and 2 were collected within the same setup of EEG. Nevertheless, the quality of collected data from studies 1 and 2 still varied for the same participant. However, the pre-processing of data was divided between studies 1 and 2 for better outcome of cleaning and excluding artifacts, and we decide whether to include each participants’ usable data according to this outcome. Therefore, the participant sets of final ERP data analysis of studies 1 and 2 are different.

#### ERP results for English words (L2)

**Figure 5:**
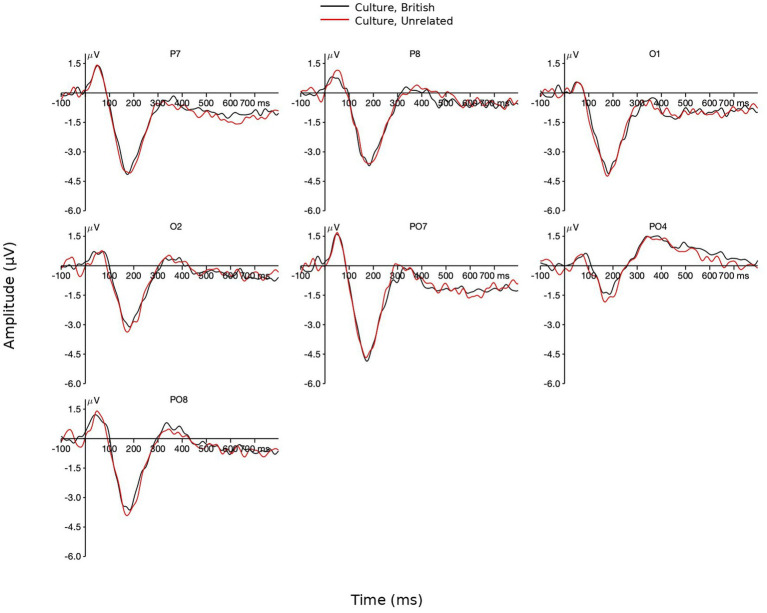
Mean ERPs for two culture conditions (British vs. unrelated) on electrodes selected for the EPN component (see data analysis in the method section) of English words. For the statistical analyses, a time window between 100 and 280 ms was used.

**Figure 6:**
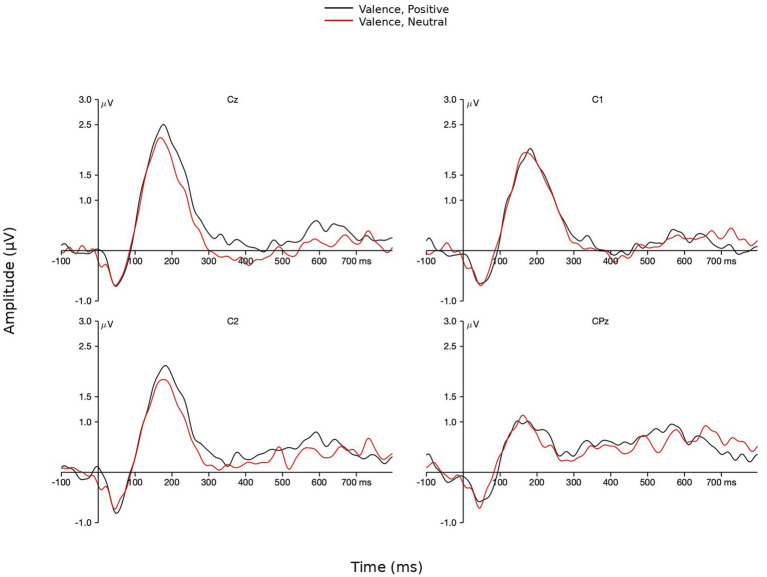
Mean ERPs for valence conditions (positive vs. neutral) on electrodes selected for the LPC component (see data analysis in the method section) of English words. For the statistical analyses, a time window between 500 and 700 ms was used.

The grand average ERP waveforms elicited by English words are illustrated in Figures 3–6, displaying the neural responses for the EPN and LPC components across the experimental conditions.

#### EPN mean amplitude

We conducted a linear mixed-effects model to investigate the effects of emotional valence, culture relatedness as well as their interations on EPN mean amplitude, with group (participant) as a random effect. The analysis included 3,746 observations from 17 participants (range = 188–237 observations per participant).

There was a significant main effect of emotional valence (positive vs. neutral), *β* = −0.48, *SE* = 0.20, *z* = −2.34, *p* = 0.019. No significant main effect of culture relatedness for the comparison between the related to British culture condition and the unrelated condition, *β* = −0.12, *SE* = 0.21, *z* = −0.57, *p* = 0.571, nor for the comparison between the related to Chinese culture condition and the unrelated condition, *β* = −0.34, *SE* = 0.20, *z* = −1.65, *p* = 0.099. The interactions (baseline: neutral valence and unrelated culture) between emotional valence and culture were not significant for either comparison (related to British culture condition: *β* = 0.35, *SE* = 0.29, *z* = 1.22, *p* = 0.221; related to Chinese culture condition: *β* = 0.46, *SE* = 0.29, *z* = 1.60, *p* = 0.110). The random intercept variance for participants was estimated at 3.03 (*SE* = 0.30). The model yielded a marginal *R^2^* of 0.002 and a conditional *R^2^* of 0.189.

#### LPC mean amplitude

We conducted a linear mixed-effects model to investigate the effects of emotional valence, culture relatedness as well as their interactions on LPC mean amplitude, with group (participant) as a random effect. The analysis included 3,746 observations from 17 participants (range = 188–237 observations per participant).

There was no significant main effect of emotional valence (positive vs. neutral), *β* = 0.26, *SE* = 0.20, *z* = 1.34, *p* = 0.180. No significant main effect of culture relatedness for the comparison between the related to British culture condition and the unrelated condition, *β* = 0.36, *SE* = 0.20, *z* = 1.81, *p* = 0.071, nor for the comparison between the related to Chinese culture condition and the unrelated condition, *β* = 0.28, *SE* = 0.20, *z* = 1.41, *p* = 0.158. The interactions (baseline: neutral valence and unrelated culture) between emotional valence and culture were not significant for either comparison (related to British culture condition: *β* = −0.47, *SE* = 0.28, *z* = −1.69, *p* = 0.091; related to Chinese culture condition: *β* = −0.24, *SE* = 0.28, *z* = −0.84, *p* = 0.399). The random intercept variance for participants was estimated at 0.89 (*SE* = 0.10). The model yielded a marginal *R^2^* of 0.001 and a conditional *R^2^* of 0.070.

#### ERP results for Mandarin words (L1)

**Figure 7:**
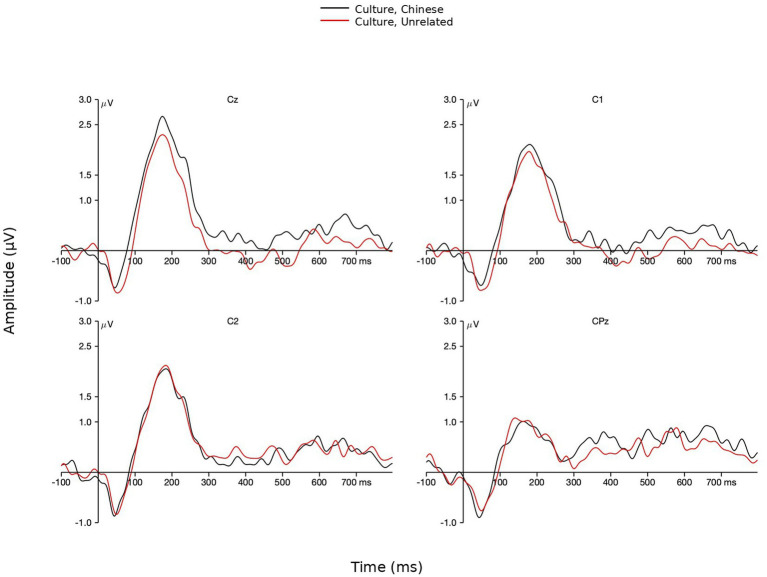
Mean ERPs for two culture conditions (Chinese vs. unrelated) on electrodes selected for the LPC component (see data analysis in the method section) of English words. For the statistical analyses, a time window between 500 and 700 ms was used.

**Figure 8:**
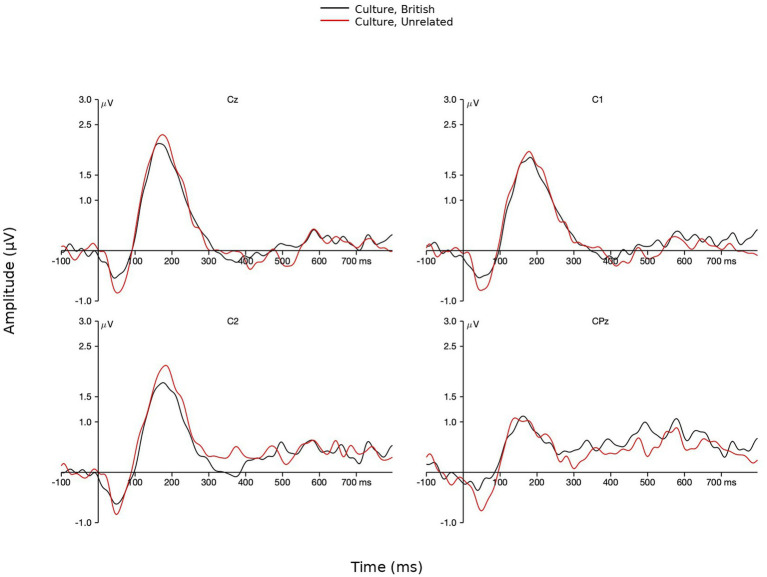
Mean ERPs for two culture conditions (British vs. unrelated) on electrodes selected for the LPC component (see data analysis in the method section) of English words. For the statistical analyses, a time window between 500 and 700 ms was used.

**Figure 9:**
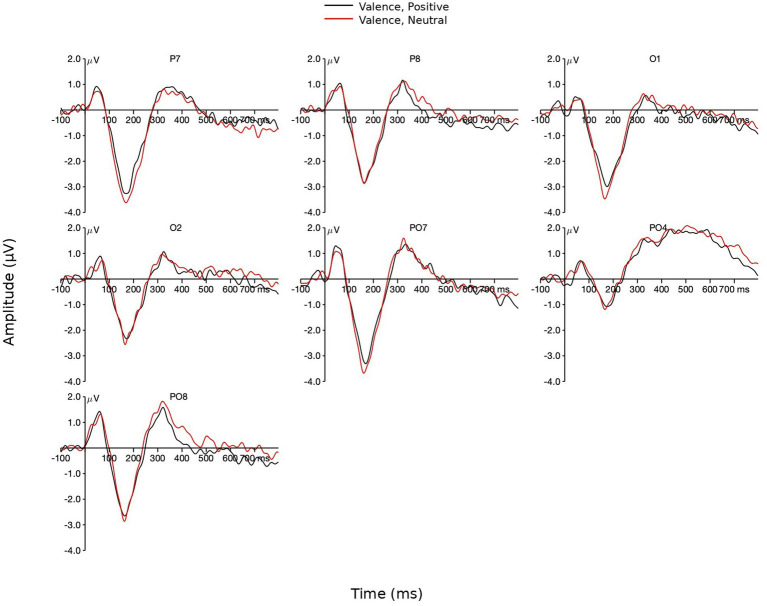
Mean ERPs for valence conditions (positive vs. neutrual) on electrodes selected for the EPN component (see data analysis in the method section) of Mandarin words. For the statistical analyses, a time window between 100 and 280 ms was used.

**Figure 10:**
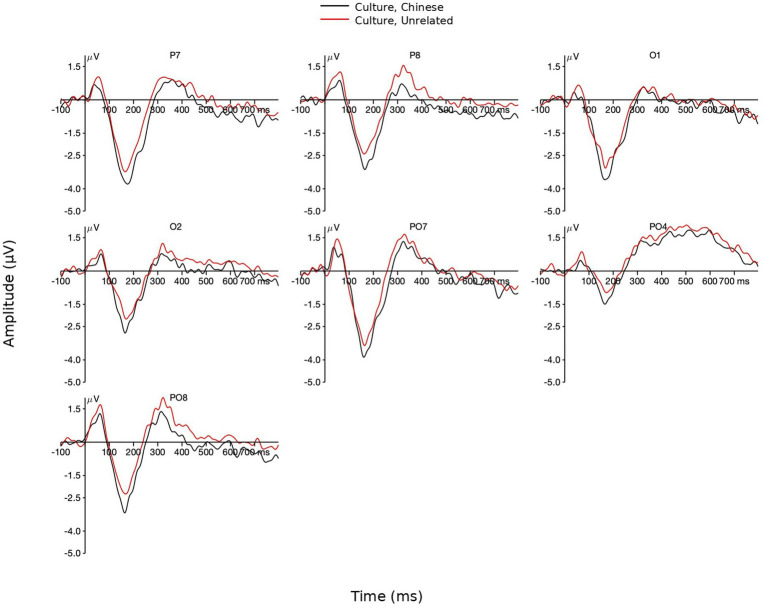
Mean ERPs for two culture conditions (Chinese vs. unrelated) on electrodes selected for the EPN component (see data analysis in the method section) of Mandarin words. For the statistical analyses, a time window between 100 and 280 ms was used.

Correspondingly, Figures 7–10 present the grand average ERP waveforms for Mandarin words, delineating the neural activity associated with the EPN and LPC components.

#### EPN mean amplitude

**Figure 11:**
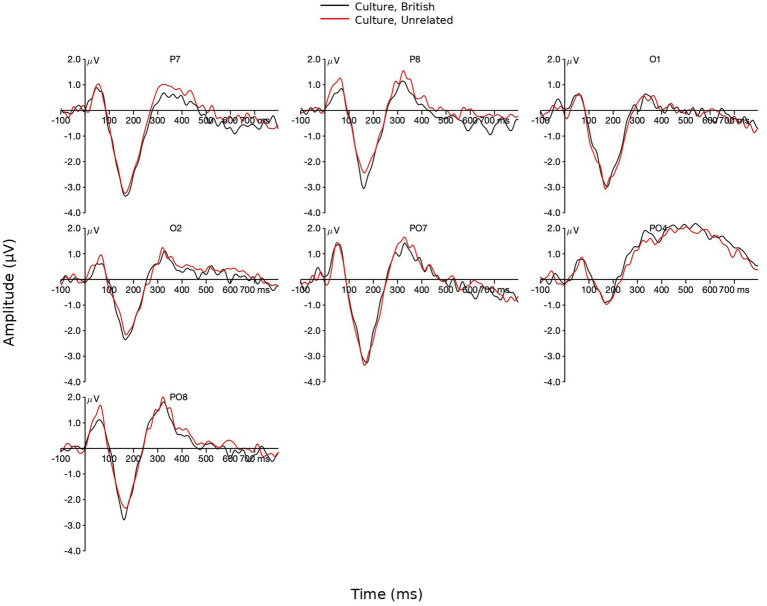
Mean ERPs for two culture conditions (British vs. unrelated) on electrodes selected for the EPN component (see data analysis in the method section) of Mandarin words. For the statistical analyses, a time window between 100 and 280 ms was used.

We conducted a linear mixed-effects model to investigate the effects of emotional valence, culture relatedness as well as their interactions on EPN mean amplitude, with group (participant) as a random effect. The analysis included 4,007 observations from 18 participants (range = 207–238 observations per group) (Figure 11).

There was no significant main effect of emotional valence (positive vs. neutral), *β* = 0.02, *SE* = 0.20, *z* = 0.07, *p* = 0.942. There was no significant main effect of culture relatedness for the comparison between the related to British culture condition and the unrelated condition, *β* = 0.08, *SE* = 0.20, *z* = 0.38, *p* = 0.702, but there was a significant main effect for the comparison between the related to Chinese culture condition and the unrelated condition, *β* = −0.55, *SE* = 0.20, *z* = −2.75, *p* = 0.006. The interactions (baseline: neutral valence and unrelated culture) between emotional valence and culture were not significant for either comparison (related to British culture condition: *β* = −0.17, *SE* = 0.28, *z* = −0.61, *p* = 0.545; related to Chinese culture condition: *β* = 0.32, *SE* = 0.28, *z* = 1.12, *p* = 0.261). The random intercept variance for participants was estimated at 2.28 (*SE* = 0.22). The model yielded a marginal *R^2^* of 0.003 and a conditional *R^2^* of 0.150.

#### LPC mean amplitude

**Figure 12:**
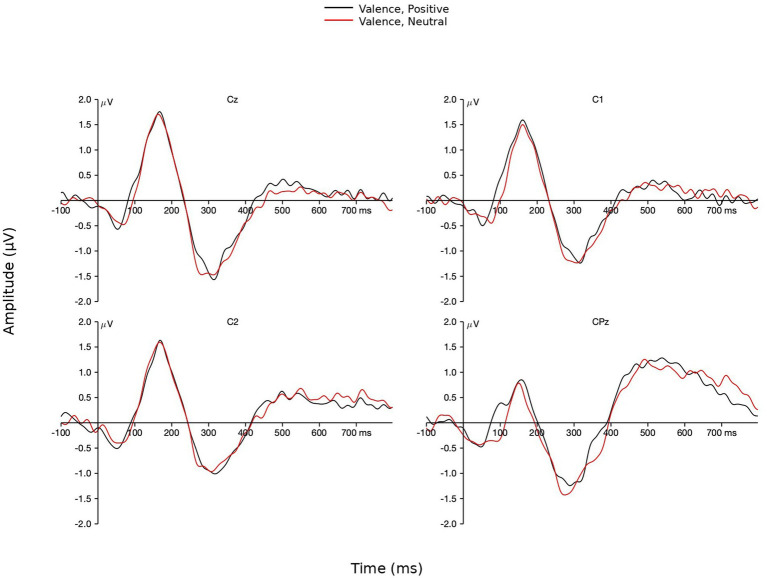
Mean ERPs for valence conditions (positive vs. neutrual) on electrodes selected for the LPC component (see data analysis in the method section) of Mandarin words. For the statistical analyses, a time window between 420 and 620 ms was used.

We conducted a linear mixed-effects model to investigate the effects of emotional valence, culture relatedness as well as their interactions on LPC mean amplitude, with group (participant) as a random effect. The analysis included 4,007 observations from 18 participants (range = 207–238 observations per group) (Figure 12).

**Figure 13:**
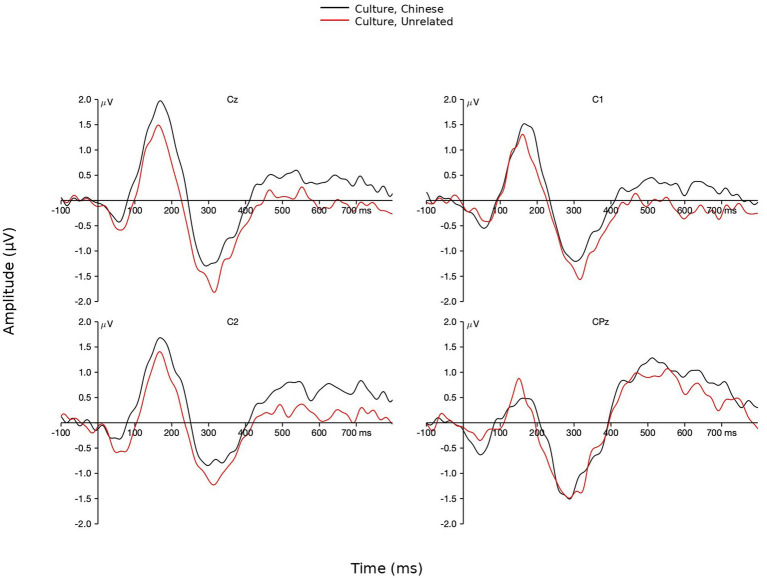
Mean ERPs for valence conditions (Chinese vs. unrelated) on electrodes selected for the LPC component (see data analysis in the method section) of Mandarin words. For the statistical analyses, a time window between 420 and 620 ms was used.

There was no significant main effect of emotional valence (positive vs. neutral), *β* = 0.30, *SE* = 0.20, *z* = 1.51, *p* = 0.132. There was a significant main effect of culture relatedness for the comparison between the related to British culture condition and the unrelated condition, *β* = 0.47, *SE* = 0.20, *z* = 2.31, *p* = 0.021, as well as for the comparison between the related to Chinese culture condition and the unrelated condition, *β* = 0.55, *SE* = 0.20, *z* = 2.75, *p* = 0.006. The interactions (baseline: neutral valence and unrelated culture) between emotional valence and culture were not significant for either comparison (related to British culture condition: *β* = −0.29, *SE* = 0.28, *z* = −1.02, *p* = 0.306; related to Chinese culture condition: *β* = −0.40, *SE* = 0.29, *z* = −1.39, *p* = 0.166). The random intercept variance for participants was estimated at 1.56 (*SE* = 0.15). The model yielded a marginal *R^2^* of 0.002 and a conditional *R^2^* of 0.106 (Figure 13).

## Discussion

**Figure 14:**
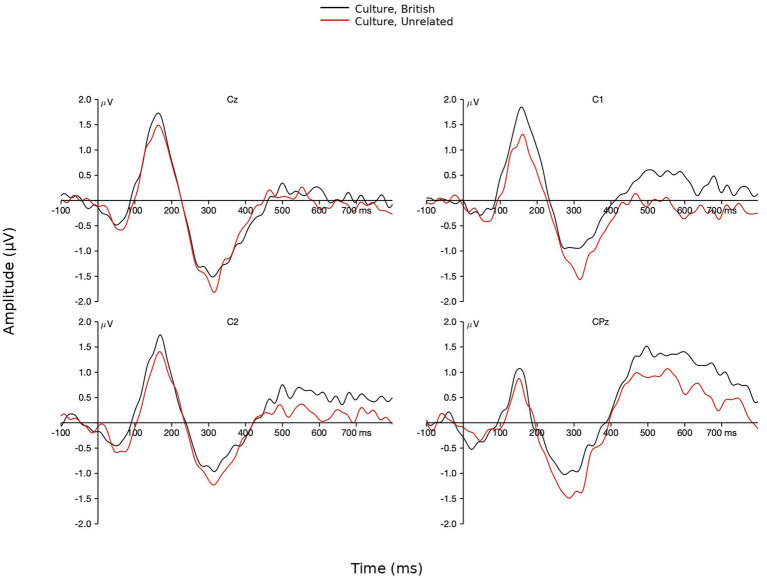
Mean ERPs for valence conditions (British vs. unrelated) on electrodes selected for the LPC component (see data analysis in the method section) of Mandarin words. For the statistical analyses, a time window between 420 and 620 ms was used.

The present study systematically investigated the interplay between language, culture, and emotion in bilingual speakers. Specifically, we examined how culture relatedness of one’s native or second language culture modulates affective processing in both the L1 (Mandarin) and the L2 (English). By employing event-related potentials (ERPs) alongside behavioral measures, we explored if cultural context influences the “emotional distance” often observed in L2 processing and how it interacts with the emotional valence of words (Figure 14).

Behavioral results consistently revealed a positivity advantage, with shorter reaction times for positive words compared to neutral ones in both the L1 and L2. However, cultural context interacted with valence: in both languages, positive words related to specific cultures (British or Chinese) were generally judged more slowly than unrelated positive words. In the L2, we also observed a general facilitation for British-culture-related words compared to unrelated words.

The behavioral findings show the well-established positivity superiority effect, suggesting that positive valence facilitates lexical processing regardless of language status (e.g., Citron, 2012; Sereno et al., 2015). The interaction between culture and valence, however, indicates that cultural salience may introduce additional cognitive processing demands during valence judgments. The unexpected finding that L2 words related to British culture were processed faster than unrelated words may reflect the participants’ immersion in the UK environment, which could have increased the accessibility of concepts that were congruent with their current cultural and daily experience, consistent with broader accounts of cognitive restructuring in bilingualism and second-language immersion (Athanasopoulos, 2011; Bylund and Athanasopoulos, 2014).

Regarding the ERP results, a robust body of literature has established that emotional valence typically modulates both the EPN and LPC components, with positive (and negative) words eliciting larger amplitudes than neutral ones (e.g., Citron, 2012; Hinojosa et al., 2010). Partially in line with these findings, we observed a significant main effect of emotional valence on the EPN component for L2 English words, which may indicate that the present stimuli were sufficiently affective to elicit early-stage affective processing in the second language.

However, contrary to standard expectations, this valence effect was notably absent in the L1 Mandarin EPN data, and absent in the late stage of affective processing indicated by LPC component for both languages. This absence of valence effects, particularly in the late time window (LPC), likely stems from specific methodological constraints in our stimulus selection. In order to manipulate culture relatedness while matching words on other psycholinguistic properties (e.g., familiarity, imageability, and length), we had to select positive words that were only moderately positive on the valence scale, rather than strongly positive items. Consequently, while the emotional intensity was sufficient to trigger early identification in L2 (EPN), it may have been insufficiently potent to sustain the elaborate processing and heightened attention typically reflected in the LPC component.

This limitation may reflect a broader challenge in investigating cultural dimensions of emotion: culturally specific concepts (e.g., scones, hot pot) may carry subtler or less uniform affective associations than the more universal emotion words (e.g., love, happy) that are often used to elicit robust ERP effects. In selecting our stimuli, we drew primarily on widely used normative word databases (i.e., Xu et al., 2022, for Mandarin and Warriner et al., 2013, for English). However, because culturally relevant words were relatively sparse in these general lists, we supplemented them with culturally distinctive items selected based on common cultural knowledge (e.g., Confucius, Princess Diana). To validate these additions, we conducted extensive pre-screening with native speakers of British English and Mandarin Chinese to ensure accurate cultural categorization. Despite these rigorous preparations, the necessity of balancing cultural and linguistic factors seemingly came at the cost of valence intensity.

Taken together, the behavioral and ERP findings suggest that culture-relatedness might have influenced affective processing at distinct stages. Notably, the culture-by-valence interaction identified in the behavioral data was not observed in the ERP results. Behaviorally, in both languages, responses were significantly slower when they were related to either British or Chinese culture than when they were unrelated to either culture. This may suggest that culture relatedness may have introduced additional demands at the stage of explicit valence categorization. In contrast, no comparable interaction between culture-relatedness and valence was observed in the EPN or LPC analyses.

One possible explanation is that the behavioral and ERP measures captured different stages of affective processing. In the L2, the overall mean response time across conditions was 1369.35 ms, whereas the LPC effect was examined within the 500–700 ms window. Similarly, in the L1, the overall mean response time was 1025.83 ms, while the LPC was examined within the 420–620 ms window. Thus, participants’ behavioral responses occurred considerably later than the ERP time windows analyzed in both studies. The behavioral culture-by-valence interaction may therefore reflect later decisional, categorization, or response-selection processes that emerged after windows of the analyzed ERP components.

We also acknowledge some key limitations from the statistical perspective. In the present dataset, the late positivity in the L2 data peaked somewhat later than in the L1 data, which motivated the use of slightly different LPC windows for the two studies. We acknowledge that this reduces strict temporal comparability across languages. One other limitation concerns the final ERP sample size. Although all 34 participants completed both studies, the usable ERP datasets were reduced after preprocessing and trial-retention procedures, leaving 17 participants in Study 1 and 18 participants in Study 2. This reduction limits statistical power, particularly for small effects and interactions, and therefore constrains the interpretation of null findings. The ERP results should therefore be interpreted cautiously and would benefit from replication in a larger sample. Another limitation pertains to the use of the LHQ data. Although LHQ responses were collected to characterize participants’ language background, they were not linked to the corresponding behavioral and event-related potential (ERP) datasets at the individual participant level. As a result, the present analyses did not evaluate whether individual differences in language history were associated with behavioral responses or ERP effects. Future research should maintain participant-level links between language background measures and experimental data to enable more direct modeling of individual variation in bilingual experience.

A further limitation is that participants made subjective valence judgments without objectively correct responses. Although this was appropriate for directing attention to the affective meaning of the stimuli, it may also have introduced additional variability due to individual differences in interpretation and categorization. More generally, emotional word effects are known to vary with task demands, including whether attention is directed explicitly to affective content (e.g., Schacht and Sommer, 2009; Citron, 2012).

Instead of the standard valence effect, the L1 processing profile was characterized by main effects of culture relatedness. Specifically, our results showed a bias toward native culture in the EPN window and a generalized sensitivity to culture relatedness to both native and second culture in the LPC window. Two key implications arise from these findings. First, given the absence of a significant interaction between culture and valence, we cannot assert that culture relatedness strictly modulated the emotional processing of the words; rather, the cultural attributes appear to have been processed independently of the words’ emotional value. Second, and perhaps more importantly, the emergence of these main effects suggests that culture relatedness plays a salient, intrinsic role in L1 word processing. Even though the experimental task directed participants’ attention specifically toward emotional valence and did not explicitly guide them to attend to cultural attributes, the brain seemingly captured and prioritized the cultural information spontaneously.

Future research is therefore needed to disentangle these mechanisms. Studies utilizing stimuli with stronger emotional valence ratings are required to successfully replicate the standard EPN/LPC valence effects within this cultural paradigm. Establishing such a clear affective baseline would allow researchers to more confidently determine whether the effects of culture relatedness are truly grounded in the same neural mechanisms that process emotional intensity.

Additionally, we noted that individuals’ actual proficiency in their second language might not be fully captured by their scores on standardized language assessments. Routine oral feedback collected after the experimental sessions revealed that some participants (native Mandarin speakers with English as L2) experienced difficulties or delays in recalling the meanings of certain English words during the task. This observation aligns with arguments from the linguistic literature, suggesting that standardized proficiency benchmarks (e.g., CEFR C1) may not reliably predict second-language users’ ability to spontaneously and accurately access semantic meanings in real-time contexts. In other words, fulfilling our recruitment criterion based on formal language proficiency did not necessarily guarantee that participants could immediately and clearly map each experimental stimulus onto the relevant experimental dimensions measured.

## Data Availability

The raw data supporting the conclusions of this article will be made available by the authors, without undue reservation.

## References

[ref1] Athanasopoulos P. (2011). “Cognitive restructuring in bilingualism,” in Thinking and Speaking in two Languages, ed. Pavlenko A. (Bristol, UK: Multilingual Matters), 29–65.

[ref4] Bylund E. Athanasopoulos P. (2014). Linguistic relativity in SLA: toward a new research program. Lang. Learn. 64, 952–985. doi: 10.1111/lang.12080

[ref7] Citron F. M. (2012). Neural correlates of written emotion word processing: a review of recent electrophysiological and hemodynamic neuroimaging studies. Brain Lang. 122, 211–226. doi: 10.1016/j.bandl.2011.12.007 22277309

[ref9] Costa A. Pannunzi M. Deco G. Pickering M. J. (2017). Do bilinguals automatically activate their native language when they are not using it? Cogn. Sci. 41, 1629–1644. doi: 10.1111/cogs.12434, 27766658

[ref10] Costa A. Santesteban M. Caño A. (2005). On the facilitatory effects of cognate words in bilingual speech production. Brain Lang. 94, 94–103. doi: 10.1016/j.bandl.2004.12.002, 15896387

[ref11] Degner J. Doycheva C. Wentura D. (2012). It matters how much you talk: on the automaticity of affective connotations of first and second language words. Bilingualism 15, 181–189. doi: 10.1017/s1366728911000095

[ref12] Delorme A. Makeig S. (2004). EEGLAB: an open source toolbox for analysis of single-trial EEG dynamics including independent component analysis. J. Neurosci. Methods 134, 9–21. doi: 10.1016/j.jneumeth.2003.10.009, 15102499

[ref14] Eilola T. M. Havelka J. Sharma D. (2007). Emotional activation in the first and second language. Cogn. Emot. 21, 1064–1076. doi: 10.1080/02699930601054109

[ref19] Han S. Northoff G. (2008). Culture-sensitive neural substrates of human cognition: a transcultural neuroimaging approach. Nat. Rev. Neurosci. 9, 646–654. doi: 10.1038/nrn2456, 18641669

[ref20] Harris C. L. Gleason J. B. Ayçiçeği A. (2006). “When is a first language more emotional? Psychophysiological evidence from bilingual speakers,” in Bilingual minds: Emotional Experience, Expression, and Representation, ed. Pavlenko A. (Clevedon, UK: Multilingual Matters), 257–283.

[ref21] Hartsuiker R. J. Pickering M. J. (2008). Language integration in bilingual sentence production. Acta Psychol. 128, 479–489. doi: 10.1016/j.actpsy.2007.08.005, 17870040

[ref22] Hinojosa J. A. Méndez-Bértolo C. Pozo M. A. (2010). Looking at emotional words is not the same as reading emotional words: behavioral and neural correlates. Psychophysiology 47, 748–757. doi: 10.1111/j.1469-8986.2010.00982.x, 20158677

[ref23] Hinojosa J. A. Moreno E. M. Ferré P. (2020). Affective neurolinguistics: towards a framework for reconciling language and emotion. Lang. Cogn. Neurosci. 35, 813–839. doi: 10.1080/23273798.2019.1620957

[ref24] Hoijer H. (Ed.) (1954). *Language in culture: Conference on the inter-relations of language and other aspects of culture*. Chicago: University of Chicago Press.

[ref26] Imbault C. Titone D. Warriner A. B. Kuperman V. (2021). How are words felt in a second language: norms for 2,628 English words for valence and arousal by L2 speakers. Biling. Lang. Cogn. 24, 281–292. doi: 10.1017/s1366728920000474

[ref27] Kramsch C. (2014). Language and culture. AILA Rev. 27, 30–55. doi: 10.1075/aila.27.02kra

[ref29] Li P. Zhang F. Yu A. Zhao X. (2020). Language history questionnaire (LHQ3): an enhanced tool for assessing multilingual experience. Biling. Lang. Cogn. 23, 938–944. doi: 10.1017/s1366728918001153

[ref30] Lopez-Calderon J. Luck S. J. (2014). ERPLAB: an open-source toolbox for the analysis of event-related potentials. Front. Hum. Neurosci. 8:213. doi: 10.3389/fnhum.2014.00213, 24782741 PMC3995046

[ref31] Morrison S. J. Demorest S. M. Aylward E. H. Cramer S. C. Maravilla K. R. (2003). FMRI investigation of cross-cultural music comprehension. NeuroImage 20, 378–384. doi: 10.1016/S1053-8119(03)00300-8, 14527597

[ref32] Mullen T. (2012). CleanLine EEGLAB plugin. Neuroimaging Informatics Tools and Resources Clearinghouse (San Diego, CA: NITRC).

[ref33] Murata A. Moser J. S. Kitayama S. (2013). Culture shapes electrocortical responses during emotion suppression. Soc. Cogn. Affect. Neurosci. 8, 595–601. doi: 10.1093/scan/nss036, 22422803 PMC3682443

[ref34] Ng S. H. Han S. Mao L. Lai J. C. (2010). Dynamic bicultural brains: fMRI study of their flexible neural representation of self and significant others in response to culture primes. Asian J. Soc. Psychol. 13, 83–91. doi: 10.1111/j.1467-839x.2010.01303.x

[ref36] Oppenheim G. Wu Y. J. Thierry G. (2018). Found in translation: late bilinguals do automatically activate their native language when they are not using it. Cogn. Sci. 42, 1700–1713. doi: 10.1111/cogs.12618, 29802646

[ref37] Park D. C. Huang C. M. (2010). Culture wires the brain: a cognitive neuroscience perspective. Perspect. Psychol. Sci. 5, 391–400. doi: 10.1177/1745691610374591, 22866061 PMC3409833

[ref41] Pavlenko A. (2012). Affective processing in bilingual speakers: disembodied cognition? Int. J. Psychol. 47, 405–428. doi: 10.1080/00207594.2012.743665, 23163422

[ref43] Peirce J. Gray J. R. Simpson S. MacAskill M. Höchenberger R. Sogo H. . (2019). Psychopy2: experiments in behavior made easy. Behav. Res. 51, 195–203. doi: 10.3758/s13428-018-01193-y, 30734206 PMC6420413

[ref44] Pion-Tonachini L. Kreutz-Delgado K. Makeig S. (2019). ICLabel: an automated electroencephalographic independent component classifier, dataset, and website. NeuroImage 198, 181–197. doi: 10.1016/j.neuroimage.2019.05.026, 31103785 PMC6592775

[ref45] Russell J. A. (1980). A circumplex model of affect. J. Pers. Soc. Psychol. 39, 1161–1178. doi: 10.1037/h0077714

[ref46] Russell J. A. (2003). Core affect and the psychological construction of emotion. Psychol. Rev. 110, 145–172. doi: 10.1037/0033-295X.110.1.145, 12529060

[ref47] Schacht A. Sommer W. (2009). Time course and task dependence of emotion effects in word processing. Cogn. Affect. Behav. Neurosci. 9, 28–43. doi: 10.3758/CABN.9.1.28, 19246325

[ref48] Sereno S. C. Scott G. G. Yao B. Thaden E. J. O’Donnell P. J. (2015). Emotion word processing: does mood make a difference? Front. Psychol. 6:1191. doi: 10.3389/fpsyg.2015.01191 26379570 PMC4547016

[ref49] Svanes B. (1987). Motivation and cultural distance in second-language acquisition. Lang. Learn. 37, 341–359. doi: 10.1111/j.1467-1770.1987.tb00575.x

[ref50] Thierry G. Wu Y. J. (2007). Brain potentials reveal unconscious translation during foreign-language comprehension. Proc. Natl. Acad. Sci. 104, 12530–12535. doi: 10.1073/pnas.0609927104, 17630288 PMC1941503

[ref52] Toivo W. Scheepers C. (2019). Pupillary responses to affective words in bilinguals’ first versus second language. PLoS One 14:e0210450. doi: 10.1371/journal.pone.0210450, 31013266 PMC6478288

[ref53] Warriner A. B. Kuperman V. Brysbaert M. (2013). Norms of valence, arousal, and dominance for 13,915 English lemmas. Behav. Res. Methods 45, 1191–1207. doi: 10.3758/s13428-012-0314-x, 23404613

[ref54] Wright D. A. (2000). Culture as information and culture as affective process: a comparative study. Foreign Lang. Ann. 33, 330–341. doi: 10.1111/j.1944-9720.2000.tb00610.x

[ref56] Xu X. Li J. Chen H. (2022). Valence and arousal ratings for 11,310 simplified Chinese words. Behav. Res. Methods 54, 26–41. doi: 10.3758/s13428-021-01607-4, 34100200

[ref58] Zhang S. Morris M. W. Cheng C. Y. Yap A. J. (2013). Heritage-culture images disrupt immigrants’ second-language processing through triggering first-language interference. Proc. Natl. Acad. Sci. 110, 11272–11277. doi: 10.1073/pnas.1304435110, 23776218 PMC3710819

